# Robotic abdominal resection of tailgut cysts – A technical note with step‐by‐step description

**DOI:** 10.1111/codi.16088

**Published:** 2022-02-23

**Authors:** Alejandro Solís‐Peña, Lena W. S. Ngu, Miquel Kraft Carré, María José Gomez Jurado, Francesc Vallribera Valls, Gianluca Pellino, Eloy Espin‐Basany

**Affiliations:** ^1^ Colorectal Unit Vall d’Hebron University Hospital Barcelona Spain; ^2^ 6072 Department of Colorectal Surgery Northumbria Healthcare NHS Foundation Trust North Shields UK; ^3^ Department of Advanced Medical and Surgical Sciences Università degli Studi della Campania “Luigi Vanvitelli” Naples Italy

**Keywords:** retrorectal tumours, robotic approach, Tailgut cysts

## Abstract

**Aim:**

Here, we describe a step‐by‐step standardized technique for tailgut cyst resection using a single‐docking robotic approach.

**Method:**

Each step of the technique is illustrated using a composite collection of four operative patient videos to demonstrate the advantages and feasibility of this technique. The robot platform utilised is Da Vinci Xi.

**Results:**

Five female patients have undergone this operation in our unit. The size of tumours ranged from 12 to 45 mm. Median operating time was 100 min (range 90–150). Mean blood loss in all the patients was less than 50 ml. There were no major intraoperative complications. One patient had a postoperative presacral collection which required radiological drainage. Length of stay in all patients was one day.

**Conclusions:**

This technique using a single‐docking robotic approach appears safe and feasible. The robotic approach results in improved dexterity and more accurate dissection, better retraction and excellent vision which improves the ease of operating in the pelvis. Therefore, this approach can be replicated for use in a wide variety of patients with tailgut cysts.

## INTRODUCTION

Tailgut cysts are rare tumours located in the retrorectal space which is bounded by the rectum and mesorectal fascia anteriorly, peritoneal reflection superiorly, presacral fascia posteriorly, iliac vessels and ureters laterally and rectosacral and Waldeyer's fascia inferiorly [1, 2, 3]. These tumours histologically can be diverse, varying from benign to malignant due to the presence of multiple embryological remnants, of which the tail gut cyst is the most common. Tailgut cysts are asymptomatic in 50% of patients with other patients experiencing symptoms related to mass effect. It is three times more common in women compared to men. The standard of treatment is surgical resection due to a risk of local complications due to mass effect and risk of malignant transformation. Various methods of surgical resection have been described including anterior approach through the abdomen, posterior approach or a combined anterior and posterior approach depending on the size of the cyst [4]. Traditionally, open surgery was performed but a minimally invasive approach is advocated as it results in a shorter length of stay and quicker patient recovery. Previously, the level of the tumour above S3 was thought to be a criterion for the anterior approach. However, there have been reported case series of patients with tumours below the level of S4 undergoing robotic assisted anterior approach above the levator muscles with good results and low postoperative morbidity. The size of the tumours has been considered an important feature in some series, with no difference between open and minimally invasive surgery [4, 5, 6].

## METHOD

We present our case series of five patients who underwent robotic abdominal resection of a tailgut cyst via the anterior approach with a step‐by‐step video showing the technique using the da Vinci Xi^®^ surgical system [7]. Patient data was collected prospectively between June 2020 and February 2021.

### Patient positioning

The patient is placed in modified Lloyd Davies position.

### Port placement

The anatomical landmarks ‐ pubic symphysis, xiphoid process, costal margins and anterior‐superior iliac spines are identified. A line is drawn between the pubic symphysis and xiphoid process. Midclavicular lines are drawn at a distance of 6–8 cm from midline. A curved line between the umbilicus and both iliac spines is created which demarcates the line at which the trocars will be placed. Pneumoperitoneum is formed using a Veress needle at Palmer's point and achieved with carbon dioxide insufflation at pressures of 12–15 mmg Hg. Trocars are placed at the spinal umbilical line between 6–8 cm apart depending on the body habitus. R3 – camera trocar is inserted 3–4 cm to the right of the midline; R2 – bipolar forceps is inserted 3–4 cm left of the midline; R1 – tip up fenestrated grasper is inserted 6–8 cm left to R2; R4 – monopolar curved scissors or needle driver is inserted 6–8 cm right of R3. The 8 mm assistant trocar is inserted 5 cm cranially and laterally from junction of trocar line and right midclavicular line.

### Surgical steps

The patient is placed in Trendelenburg position with a right‐side tilt and manual displacement of small bowel and greater omentum towards the upper abdomen is performed.

### Docking

The robot is positioned on the left side of the patient at a 90° angle. The robot arms are aligned with the trocars and targeting performed. Robotic instruments are introduced under direct vision.

### Lateral mobilization of the rectum

The tip up grasper in R1 is used to retract the sigmoid colon cranially and laterally to expose the sacral promontory. Peritoneal dissection is performed anterior to the sacral promontory ensuring that the left common iliac vein, median sacral vessels, right hypogastric nerves and right ureter are clearly identified and protected. Bipolar forceps provides counter traction of the peritoneum with monopolar‐curved scissors used for dissection which begins at the level of the sacral promontory and continues along the right border of the mesorectum. This allows for right lateral mobilization of the rectum down to the pelvic floor and adequate exposure of the perineal body. Continuous adjustment to traction is performed to expose the correct planes.

### Pelvic dissection

Once the tumour is identified, care must be taken when separating the tumour from the posterior rectum to avoid damage or perforation. Dissection is carefully performed whilst maintaining adequate traction when mobilising the cyst to avoid tumour perforation. After the cyst is fully mobilised, washout of the surgical bed is performed and haemostasis confirmed. The specimen is extracted via a bag through a small Pfannenstiel incision, and the trocars are removed under direct vision.

## COMPARISON WITH OTHER METHODS, ADVANTAGES, AND DISADVANTAGES

Other surgical approaches described in the literature include the anterior, posterior, or combined approach depending on the size and location of the tumour [4]. The combined approach is preferred when there is nerve involvement as it allows for improved visualization of ureters, vessels, pelvic nerves, and rectum in the anterior approach and good exposure of the nerve roots provided by the posterior approach [8, 9]. However, the posterior approach is also associated with a risk of injury to lateral pelvic nerves or haemorrhage [10]. A posterior approach is more commonly used for lesions distal to S3, and most patients require resection of the coccyx, which can prolong the length of stay and cause chronic pain. Different minimally invasive approaches have been described including laparoscopic, robotic and transanal minimally invasive surgery (TAMIS). TAMIS is associated with a higher risk of pelvic infection [11]. Laparoscopic or robotic surgery with anterior approach allows for enhanced visualization of pelvic structures and precise dissection. Robotic surgery specifically provides three‐dimensional views, superior dexterity with multiarticulated instruments and good retraction [5]. Tumours located close to levator ani and coccygeal muscles are easily accessible and if there is an injury to the surrounding organs, repair is often much easier with this approach [12].

## RESULTS

We describe data from five female patients. Their symptoms ranged from abdominal pain or proctalgia to asymptomatic patients with incidental findings. Demographic and operative details of the patients are listed in Table 1. There were no major complications. Histology in all the patients confirmed retrorectal cystic hamartomas. An intraoperative air‐leak test was performed in all cases and no rectal injuries were detected. Mean length of stay was one day.

**TABLE 1:** Patient characteristics (*n* = 5)

Age (years)	49 (range 39–68)
Gender	Female
Body mass index (kg/m^2^)	26 (range 21–30)
Tail gut cyst	5
Size of the tumour (mm)	35 × 22 × 21 (range 12–45)
Symptoms	Nonspecific abdominal pain (2 patients) Incidental finding on endometriosis MRI (1 patient) Pain at the coccyx (1 patient) Proctalgia and defaecation urge (1 patient)

The postoperative discomfort of the patients was minimal in two patients, two patients had no postoperative pain, and one patient reported the same preoperative discomfort (Table 2). One patient was diagnosed with presacral collection a month after surgery, which required radiology guided drainage.

**TABLE 2:** Surgical details and postoperative outcome

Median duration of surgery	100 min (range 90–150)
Blood loss	<50 ml (all patients)
Intraoperative complications	Tumour perforation (2 patients)
Length of stay	24 h (all patients)
Postoperative pain	Mínimal discomfort in the sacral and perineum region (2 patients) No pain (2 patients) Same preoperative discomfort (1 patient)
Postoperative complications	One patient: 5 × 4 cm presacral collection

## CONCLUSION

This technique seems safe and feasible and might be adopted as an alternative when the surgeon is experienced in minimally invasive surgery especially if the cysts are located above the levator muscles. Studies with larger samples are necessary to confirm the outcomes of this technique against other surgical approaches.

## CONFLICT OF INTEREST

The authors declare that they have no conflict of interests.

## AUTHOR CONTRIBUTIONS

EEB, ASP and GP conceived the study. ASP, LWSN, MJGJ, MKC collected data and revised the literature. ASP, LWSN, GP drafted the article. FVV and EEB critically revised the article. All authors approved the final version.

## ETHICAL APPROVAL

All patients gave written informed consent before undergoing the procedure and for recording and publication. This study was performed in compliance with the Declaration of Helsinki.

## Supporting information

Video S1

## Data Availability

The data that support the findings of this study are available on request from the corresponding author. The data are not publicly available due to privacy or ethical restrictions.

## References

[1] Canelles E , Roig JV , Cantos M , García Armengol J , Barreiro E , Villalba FL , et al. Tumores presacros. Análisis de nuestra experiencia en 20 casos tratados quirúrgicamente [Presacral tumors. Analysis of 20 surgically treated patients]. Cir Esp. 2009;85(6):371–7. Spanish. 10.1016/j.ciresp.2009.01.007 19423088

[2] Lorenzo‐Liñán MA , Roig JV , Martínez E , Reina A , García‐Armengol J . Perineal hernia of a retrorectal tumour. Colorectal Dis. 2011;13(3):e46–7. 10.1111/j.1463-1318.2010.02479.x 20977589

[3] García‐Armengol J , García‐Botello S , Martinez‐Soriano F , Roig JV , Lledó S . Review of the anatomic concepts in relation to the retrorectal space and endopelvic fascia: Waldeyer's fascia and the rectosacral fascia. Colorectal Dis. 2008;10(3):298–302. 10.1111/j.1463-1318.2007.01472.x 18257849

[4] Baek SK , Hwang GS , Vinci A , Jafari MD , Jafari F , Moghadamyeghaneh Z , et al. Retrorectal tumors: a comprehensive literature review. World J Surg. 2016;40(8):2001–15. 10.1007/s00268-016-3501-6 27083451

[5] Mullaney TG , Lightner AL , Johnston M , Kelley SR , Larson DW , Dozois EJ . A systematic review of minimally invasive surgery for retrorectal tumors. Tech Coloproctol. 2018;22(4):255–63. 10.1007/s10151-018-1781-6 29679245

[6] Rompen IF , Scheiwiller A , Winiger A , Metzger J , Gass JM . Robotic‐assisted laparoscopic resection of tailgut cysts. JSLS. 2021;25(3):e2021.00035. 10.4293/JSLS.2021.00035 PMC8325480 34354334

[7] Celentano V , Smart N , McGrath J , Cahill RA , Spinelli A , Challacombe B , et al. How to report educational videos in robotic surgery: an international multidisciplinary consensus statement. Updates Surg. 2021;73(3):815–21. 10.1007/s13304-020-00734-5 32146699 PMC8184705

[8] Pellino G , García‐Granero A , Fletcher‐Sanfeliu D , Navasquillo‐Tamarit M , Frasson M , García‐Calderon D , et al. Preoperative surgical planning based on cadaver simulation and 3D imaging for a retrorectal tumour: description and video demonstration. Tech Coloproctol. 2018;22(9):709–13. 10.1007/s10151-018-1854-6 30225754

[9] Hobson KG , Ghaemmaghami V , Roe JP , Goodnight JE , Khatri VP . Tumors of the retrorectal space. Dis Colon Rectum. 2005;48(10):1964–74. 10.1007/s10350-005-0122-9 15981068

[10] Ghosh J , Eglinton T , Frizelle FA , Watson AJ . Presacral tumours in adults. Surgeon. 2007;5(1):31–8. 10.1016/s1479-666x(07)80109-0 17313126

[11] Haydar M , Griepentrog K . Tailgut cyst: a case report and literature review. Int J Surg Case Rep. 2015;10:166–8. 10.1016/j.ijscr.2015.03.031 25853843 PMC4430219

[12] Cataneo J , Cataldo T , Poylin V . Robotic excision of retrorectal mass. J Gastrointest Surg. 2018;22(10):1811–3. 10.1007/s11605-018-3838-2 29907936

